# Effects of Dietary Polyunsaturated Fatty Acids on DNA Methylation and the Expression of *DNMT3b* and *PPARα* Genes in Rats

**Published:** 2018

**Authors:** Ehsan Maktoobian Baharanchi, Mostafa Moradi Sarabi, Fakhraddin Naghibalhossaini

**Affiliations:** 1. Department of Biochemistry, Faculty of Medicine, Shiraz University of Medical Sciences, Shiraz, Iran; 2. Autoimmune Research Center, Faculty of Medicine, Shiraz University of Medical Sciences, Shiraz, Iran

**Keywords:** DNA methylation, Gene expression, Dietary supplement regulation, Fatty acids omega-3

## Abstract

**Background:**

Previous studies have suggested a protective role for Polyunsaturated Fatty Acids (PUFA) against cancer, cardiovascular, and other diseases. To provide new insights into the *in vivo* effects of PUFA on gene expression, the effects of dietary PUFA on *DNMT3b* and *PPARα* gene expression and global DNA methylation were investigated in selected rat tissues.

**Methods:**

Thirty sprague-dawley rats were allotted into 3 dietary groups of ten animals each, received experimental diets containing PUFAs every day by gavages for 12 weeks as follows: control group fed a normal diet and water; n-3 PUFAs group received 300 *mg/kg/day* n-3 PUFAs supplementation; mixed-PUFAs group received 300 *mg/kg/day* of a mixture of n-3, -6, -9 PUFAs supplementations. The expressions of *DNMT3b* and *PPARα* genes were quantitated using real-time RT-PCR. The genome-wide 5-methylcytosine contents in rat tissues were determined by ELISA method.

**Results:**

The average expression of the *DNMT3b* mRNA was 50% lower in the colon and liver of rats fed the n-3- or mixed-PUFAs supplemented diet than control group (p=0.00). However, *PPARα* expression was significantly upregulated both in the colon and liver of PUFAs-supplemented rats (p<0.001). No significant difference was observed in the blood, colon, and liver DNA methylation levels between PUFAs-supplemented and control animals.

**Conclusion:**

The results indicate that dietary PUFAs could modulate the expressions of *PPARα* and *DNMT3b* genes in various rat tissues. The findings of this study provide additional insights into the *in vivo* mechanism of PUFA-mediated regulation of gene expression and could provide an opportunity to develop personalized diets for related disease control.

## Introduction

Nutrigenomics is a well-established field of research that aims to find nutritional influences on gene expression. Exposure to environmental factors such as diet could induce epigenetic changes that lead to altered gene expression 1,2. Omega (ω, n)-3 Polyunsaturated Fatty Acids (PUFAs), a component of marine oils has been implicated in the prevention of cardiovascular disease, cancer, type 2 diabetes mellitus, and neurodegenerative diseases in humans 3–5. There is also evidence suggesting a protective role for n-3 PUFA supplementation in the prevention of Colorectal Cancer (CRC) 3,6. Administration of n-3 PUFAs in both rodent models of CRC and humans has been demonstrated to increase n-3 fatty acids content of tumors and colonic mucosa, respectively 7. Other studies have reported significant reduction of the size of xenograft tumors of human CRC cell lines in rodents supplemented with dietary PUFAs as compared to controls 8,9.

Numerous mechanisms have been suggested by which n-3 PUFAs might suppress cancer cell growth, including regulation of gene expression, cell migration, angiogenesis, and apoptosis 10. It has been well-documented that fatty acids, especially PUFAs can regulate genes expression through binding to the intracellular peroxisome Proliferator-Activated Receptors (PPARs) 11. Since many ligands for PPARs, like NSAIDs, have been shown to inhibit tumor cell proliferation 12,13, it has been hypothesized that n-3 PUFAs might exert their antineoplastic activity through differential activation of PPAR-α and PPAR-γ receptors 14. However, data are inconsistent, and little is known about the exact mechanisms by which PPAR activation prevents carcinogenesis 15–17.

Aberrant global DNA hypomethylation and CpG Island DNA hypermethylation are the most common epigenetic alterations observed in CRC tumors 18. Clinical and experimental studies indicated that expression of DNA methyltransferases especially that of *DNMT3B* could contribute to aberrant DNA methylation in CRC tumors 19–21. There are emerging findings indicating that n-3 PUFA treatment can modulate epigenome in cells 22. Such epigenetic changes likely play an important role in the mechanisms involved in the observed n-3 PUFA effects on gene expression. These studies have indicated that PUFAs exposure might modulate gene specific and global DNA methylations as well as histone modifications that are consistent with changes to gene expression 23–26. In humans, maternal supplementation with n-3 PUFA during pregnancy may modify global DNA methylation levels in infants 23. In rodents, dietary *n*-3 PUFA supplementation during pregnancy or lactation has been reported to induce promoter methylation of *Fads2* gene and reduce its mRNA expression in maternal and offspring livers compared to those fed soy bean oil 27. In the present study, the effect of PUFAs supplementation on the expression of *DNMT3b* and *PPARα* genes and global DNA methylation was investigated in selected normal rat tissues.

## Materials and Methods

### Animal procedures

Thirty sprague-dawley rats, 3 weeks old and weighing 160–250 *gr* were used for this study. Rats were housed at 5 animals per cage and kept in 12h light-dark cycles. All animals had free access to the regular laboratory food pellets (Behparvar, Tehran) containing 3.5–4.5% total lipid and tap water. All protocols were approved by the institutional animal care committee of Shiraz University of Medical Sciences. The rats were divided into 3 dietary groups of 10 (5 females and 5 males/each group) as follows: group 1 (control group) received water every day by gavage for 12 weeks; group 2 (n-3 PUFA group) received 300 *mg/kg/day* n-3 PUFA (Docosahexaenoic acid, DHA: 120 *mg/kg*+ Eicosapentaenoic acid, EPA: 180 *mg/kg*) (Golden Alaska Deep Sea Fish Oil, USA) per day by oral gavage for 12 weeks; group 3 (mixed-PUFA group) received 300 *mg/kg/day* of a mixture of n-3, -6, -9 [(DHA: 20 *mg/kg*+EPA: 30 *mg/kg*+Linoleic acid, LA: 75 *mg/kg*+α-linolenic acid, α-LNA: 83.4 *mg/kg*+γ-Linolenic acid, γ-LNA: 33.3 *mg/kg*+Oleic acid, OA: 58.3 *mg/kg*) (Nutralife, Numega, Canada)] by gavage for 12 weeks. After treatment period, rats were sacrificed and whole blood was collected by cardiac puncture and sections of colon and liver were then surgically removed, frozen in liquid nitrogen, and stored at −80*°C* until needed.

### DNA extraction and global DNA methylation analysis

High molecular weight genomic DNA was isolated from blood, liver, and colon tissues by the standard protocol of proteinase K digestion and phenol-chloroform extraction. Global DNA methylation was quantified using 5-mC DNA ELISA kit (Zymo Research, Germany), as described previously 28.

### RNA extraction and quantitative RT-PCR

Total RNA was extracted from colon and liver tissues using Tripure RNA isolation reagent (Roche Applied Science, Germany), following the manufacturer’s instructions. Purified RNA was stored at −80*°C* until use. Complementary DNA (cDNA) was prepared from each RNA sample as described previously 29. The expression levels of genes of interest (*DNMT3b* and *PPARα*) and reference gene (*β-actin*) were determined by quantitative real-time RT-PCR using SYBR green-based analysis and Master Mix (ABI, UK). The sequences of primers used for amplification of genes are listed in table 1. To prevent nonspecific amplification of the possible contaminating genomic DNA, the forward and reverse primers were used for RT-PCR amplification of *DNMT3b* and *β-actin* genes, designed on different exons with a large intron between them. Reactions were carried out in triplicate and analyzed using an ABI 7500 Sequence Detection System (Applied Biosystems, USA). Amplifications were performed under the following conditions: a pre-cycling heat activation at 95°*C* for 10 *min*, followed by 38 cycles of heat denaturation at 95°*C* for 15 *s* and annealing and extension at 60*°C* for 1 *min*. Relative expression levels were determined using the standard ΔCt method with *β-actin* internal reference gene used for normalization 30.

### Statistical analysis

The data are presented as the mean±SD and SPSS18 analytic software (SPSS, Inc., Chicago) and GraphPad Prism statistical software (version 5; San Diego, CA) were used for data analysis. One-way ANOVA with Tukey’s post hoc test were used to determine differences between groups, as indicated. The significance level was set at p< 0.05.

**Table 1.** Primers’ sequence used for quantitative RT-PCR

**Gene**	**Forward primer (5′ → 3′)**	**Reverse primer (5′→ 3′)**	**Target size (*bp*)**
***DNMT3b***	GATGATCGACGCCATCAAG	CGAGCTTATCATTCTTTGAAGCTA	107
***PPARα***	TGAACAAAGACGGGATG	TCAAACTTGGGTTCCATGAT	106
***β-actin***	AAGGCCAACCGTGAAAAGAT	ACCAGAGGCATACAGGGACA	102

## Results

### Effect of dietary PUFA on the expression of DNMT3b and PPARα genes in rats

To assess the influence of dietary PUFA supplementation on the regulation of *DNMT3b* and *PPARα* genes expression *in vivo*, 2 groups of rats were fed daily either with 300 *mg/kg* n-3 PUFA (DHA+EPA) (n-3 PUFAs group) or a mixture of n-3, -6, -9 PUFAS (mixed PUFAs group) for 3 months as described in the Material and Methods section. *DNMT3b* and *PPARα* genes expression in the colon and liver tissues was measured and compared with those in the control group (animals on a normal diet). The average expression levels were calculated from the combined expression values for each group (n=10) and are presented as mean±SD.

**Figure 1.**
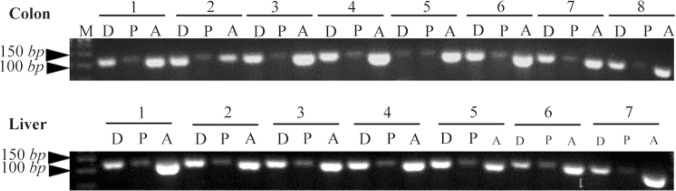
Agarose gel electrophoresis of RT-PCR products of *DNMT3b*, *PPARα* and *β-actin* gene in the colon and liver tissues of rats. The presence of PCR products with the expected lengths of about 107 *bp* for *DNMT3b*, 106 *bp* for *PPARα* and 102 *bp* for *β-actin* was confirmed. M, DNA marker; D, *DNMT3b*; P, *PPARα*; A: *β-actin*.

Before quantitation, electrophoresis of RT-PCR pro-ducts on 1.5% agarose gel and visualization under UV illumination confirmed that specific products of about 107, 106, and 102 *bp* with no non-specific PCR products were obtained upon amplification of *DNMT3b*, *PPARα*, and *β-actin* cDNAs, respectively (Figure 1). To ensure there was no amplification of contaminating genomic DNA, minus RT control PCR reactions were performed in which no reverse transcriptase was added to RNA samples. No amplification product was detected for any specific pair of primers used in these reactions.

**Figure 2.**
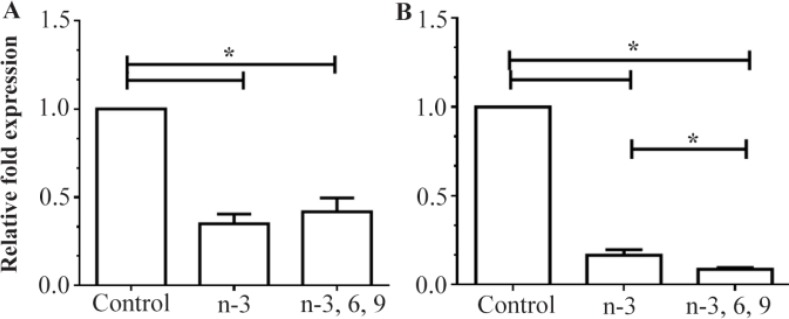
Relative expression of *DNMT3b* in A) liver and B) colon tissue of rats fed with the n-3 PUFA and mixed PUFA supplemented diets measured by quantitative RT-PCR. The mean expression of each gene was normalized to *β-actin* mRNA. Water fed groups were used as controls, whose expression levels were set to 1.0, and expressions of n-3 PUFA and mixed PUFA supplemented groups were expressed as an n-fold difference relative to the control group. Mean values±SEM of three experiments are given. Bars marked with asterisk are significantly different as verified by Tukey’s honestly significant difference multiple comparison test (p<0.001).

**Figure 3.**
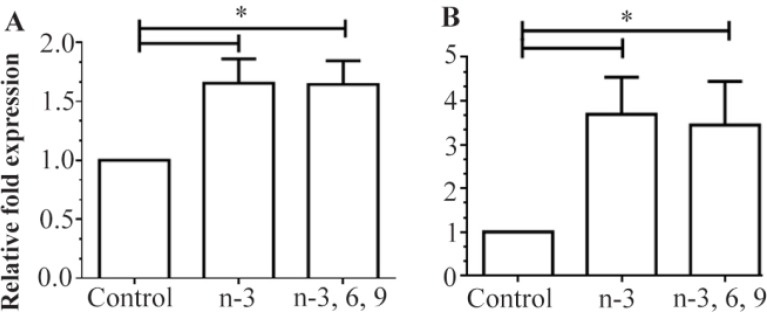
Relative expression of *PPARα* in A) liver and B) colon tissues of rats fed with the n-3 PUFA and mixed PUFAs supplemented diets measured by quantitative RT-PCR. Mean expression of each gene was normalized to *β-actin* mRNA. Water fed groups were used as a control, whose expression levels were set to 1.0, and expressions of n-3 PUFA and mixed PUFA groups were expressed as an n-fold difference relative to control group. Mean values±SEM of three experiments are given. Bars marked with asterisk are significantly different as verified by Tukey’s honestly significant difference multiple comparison test (p<0.001).

As shown in figure 2, both n-3 PUFAs and mixed PUFAs significantly decreased *DNMT3b* mRNA levels in colon and liver tissues as compared to controls (p=0.00). More than 50% reduction in *DNMT3b* expression was observed in liver tissues from the rats fed with n-3 PUFAs or mixed PUFAs (Figure 2A). The average expression levels of *DNMT3b* were also found to be 83.5% and 91.4% lower in the colon tissues of rats fed the n-3 PUFA and mixed PUFA supplemented diets, respectively (Figure 2B). However, *PPARα* expression was significantly higher in the liver and colon tissues of rats fed the n-3 PUFA and mixed PUFA supplemented diets as compared to controls (p<0.001) (Figure 3). In comparison with controls, average expression**s** of *PPARα* mRNA were 1.65- and 3.7-fold higher in the liver and colon tissues of n-3 PUFAs group, respectively (Figure 3A). The data also showed an overall about 1.6- and 3.5-fold upregulation of *PPARα* expression in the liver and colon tissues of the mixed PUFAs group as compared to controls (Figure 3B).

### Global DNA methylation analysis

**Figure 4.**
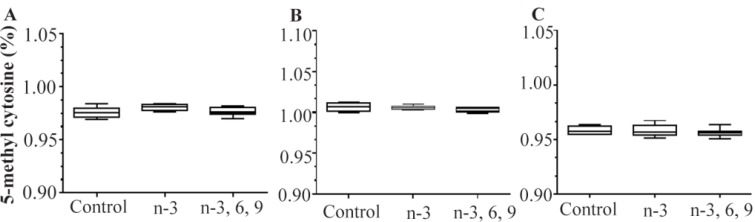
Comparison of mean global DNA methylation levels in A) colon, B) blood, and C) liver tissues between mixed PUFA, n-3 PUFA, and control animals. Percentage of 5-mC in rats was evaluated using ELISA assay. Mean values±SEM of three experiments are given (p<0.05).

Rats were supplemented with n-3 PUFAs or mixed-PUFAs for 3 months and DNA methylation levels in the blood, liver, and colorectal tissues of PUFA-supplemented animals were compared with those of controls. The average methylation levels was calculated from the combined methylation values for each group (n=10) and are presented as mean±SD (Figure 4). There was no difference in the blood, colon, and liver global DNA methylation levels between mixed PUFA, n-3 PUFA, and control groups (Figure 4). The data showed that overall about 0.976%, 0.981%, and 0.976% of the cytosines were methylated in the genomic DNA extracted from colonic tissues of mixed PUFAs, n-3 PUFAs, and control group, respectively (p>0.05) (Figure 4A). The mean global methylation percentage in DNA from blood was 1.006% for n-3 PUFA group, 1.003% for mixed PUFA group, and 1.006% for controls, a non-significant difference (p= 0.79) (Figure 4B). The mean percentage of 5-mC in the DNA extracted from liver of n-3 PUFA group, mixed PUFA group, and controls was 0.958, 0.956, and 0.958%, respectively (p>0.05) (Figure 4C).

## Discussion

Characterizing the molecular mechanism(s) by which n-3 PUFAs regulate gene regulation will provide an opportunity to develop personalized diets for the related diseases control. Previous studies have suggested that n-3 PUFA might reduce the risk of cancer development through a variety of mechanisms, including inhibition of angiogenesis, modulation of Cyclooxygenase (COX) activity, apoptosis, alterations to cell signaling, and anti-inflammation 14.

To provide further insights into the molecular mechanisms of PUFA activities, the influence of dietary PUFAs was investigated on *DNMT3b* and *PPARα* expressions in intestine and liver tissues of rats fed n-3 or mixed n-3, 6, 9 PUFAs-supplemented diet. Our findings showed that *DNMT3b* mRNA levels were significantly lower in liver and colon tissues of animals fed n-3 PUFAs and mixed PUFAs than control group (Figures 2A and B). Mixed PUFAs was more effective than n-3 PUFAs for reducing *DNMT3b* expression in rat colon (p<0.05). However, there was no statistically significant difference between n-3 PUFAs and mixed PUFAs groups in *DNMT3b* expression levels in liver tissue. To the best of our knowledge, this is the first report describing the PUFA-mediated downregulation of *DNMT3b* expression in animal tissues.

Deregulation of DNA methylation is the most common epigenetic alteration associated with human cancers 18. Several previous studies have indicated that *DNMT3b* mRNA is overexpressed in human CRC as compared with matched normal colonic mucosa 20,31. *DNMT3B* activity has been linked with aberrant methylation of CpG islands in colon cancer 31. Overexpression of *DNMT3B* has been shown to induce DNA methylation in specific genes promoter and colon tumor formation in mice 19. Other studies have suggested that DNMTs expression levels may directly influence global DNA methylation patterns 28,32. One recent study has investigated the effects of high fat diet and fish oil, rich in n-3 PUFA on DNA methylation in mice. Compared to animals fed control diet, fish oil prevented obesity induced changes in global and *Pparg2* promoter DNA methylation in a tissue-specific manner 24. It has been also reported that human maternal supplementation with n-3 PUFA during pregnancy may modulate global DNA methylation levels in infants 23. The finding of other studies has also suggested that dietary fat intake can modulate genome-wide DNA methylation in the skeletal muscle of healthy men 25,33. Such epigenetic changes may have an important role in the observed PUFA effects on gene expression.

Given the observed link between DNMTs enzyme activity and regulation of the epigenome, an attempt was made to examine whether dietary PUFA could modulate global DNA methylation in selected rat tissues. The levels of 5-mC in genomic DNA isolated from blood, colon and liver tissues of PUFA-supplemented animals were quantified. As shown in figure 4, the overall 5-mC levels were similar between PUF-As-supplemented animals and controls. DNA methylation is primarily mediated by a family of 3 DNMTs comprising DNMT1, -3A, and -3B in mammals 34. So far, only few studies have investigated the influence of PUFAs on DNA methylation in animal models, and their findings indicated that the epigenetic effect of n-3 PUFAs is gene and tissue specific 22,26,28,35. A positive correlation between mean DNMT1/3A/3B expression and global DNA methylation levels measured in human CRC cell lines was previously reported 28. In the present study, only the effect of dietary PUFAs on the expression of one of 3 DNMT enzym**es**, *DNMT3b*, was examined. Whether the intake of PUFA can also modulate the expression of two other DNMTs remains to be investigated.

PUFAs regulate the expression of genes in various tissues by directly binding to nuclear receptors, PPARs 36. *PPARα* is expressed predominantly in liver, heart, and intestine, playing a major role in lipid and carbohydrate metabolism and energy homeostasis 37. The expression of *PPARα* mRNA was previously correlated with liver and bladder cancers in rodents 38,39. To investigate whether there is an association between PUFA intake and expression of *PPARα*, the levels of *PPARα* mRNA in liver and colon of PUFA-supplemented rats were quantified and compared with those in animals on a normal diet. *PPARα* expression tended to be higher in both liver and colon tissues of PUFAs-supplemented rats compared to animals on a normal diet (p<0.001) (Figure 3). Therefore, our findings agree with the previous studies that reported an association between n-3 PUFAs supplementation and upregulation of *PPARα* expression in liver and colon tissues 40,41. There was no difference, however, in *PPARα* gene expression between n-3 PUFAs and mixed PUFAs groups in either tissue. The data suggest that PUFAs, as a natural ligand of PPARs not only increases *PPARα* activity, but they also modulate its expression in target tissues.

## Conclusion

The findings of this study suggest that dietary PUF-As modulate the expressions of *PPARα* and *DNMT3b* genes in rat colon and liver tissues. The data provide additional insights into the molecular mechanisms of n-3 PUFAs protective activity against various related diseases.
